# The Pre-Dental Year in Dental Education

**Published:** 1921-09

**Authors:** 


### THE PRE-DENTAL YEAR IN DENTAL EDUCATION

For some time there has been considerable discussion over the advisability of adding one year of college work as a requirement to enter dental school. As a result of this agitation, a number of schools, some of their own volition and others because of changes in dental laws, will begin the course of 1921 with one year of college work as a requirement for admission to dental college. We believe this is a great mistake, because the four year dental course has not been worked out to the satisfaction of many. Until dental colleges have had time to fully test the value of the four year course, the addition of one year of college work as a requirement is also more or less of an experiment. In the first place, we fail to see how one year of college work is going to be of any benefit in the study of dentistry. A student who has graduated from High School, has a sufficient knowledge of all collateral subjects to enable him to study dentistry with a degree of intelligence. It is a recognized fact that the first year in college is a year wasted by the majority of students. It takes one year before the average college student is able to find himself and to become accustomed to his surroundings so that he can do creditable work. Therefore it seems that this year of college work, which will be required by some schools for admission to dental college is a year of time absolutely lost.

Under the present conditions with the four year dental course, a student finds the time is long enough at best which is required to complete an education. The addition of the extra year simply means a loss of time to the student, an economical loss to the community, because this is one more year the student is not a producer. Therefore from an economic view point, the addition of one year of college work is wrong.

At the present time the number of men in the dental profession is not sufficient to take care of the needs of the public. With the addition of one year of college work, there will be a diminished number of men studying dentistry, with the result that there will not be enough men in a few years to take care of the public. As this fact becomes recognized there is a possibility that legislative bodies will so change the dental laws, as to allow anyone to practice dentistry regardless of the high requirements that have been set up by certain schools. This is more than possible as proved by the fact that one state has already so changed the medical laws that a graduate of any medical college, regardless of the standing that college has, will be permitted to practice. A few years ago because of the attitude which a certain state took in regard to educational matters, the State Board was revoked by the Legislative body and the dental profession of that state was thrown wide open.

It must be remembered that the great function of the dental profession is to serve the public, and whenever educational requirements are so raised by a number of enthusiasts, as to destroy the usefulness of the profession, the question of education will be taken out of the hands of a few and regulated by the masses which regulations will be detrimental to high ideals.

Another phase of the subject is the fact that the majority of men who are advocating one year of college work as a requirement for admission to dental schools, are men who are connected with Universities. This seems to be a move on the part of the so-called "University School" to establish such conditions as will render continuation of dental schools not affiliated with universities impossible. In fact under present conditions, very few universities, except those who have dental departments, are willing to give a student one year of college work, knowing that he will switch to dentistry at the end of that year. Therefore, certain dental colleges are being discriminated against by men who advocate one year of college work.

High requirements in dental schools are desirable up to a certain degree, but as soon as those requirements begin to react against the efficiency of dental education, then it is time to stop and consider the real motive. In considering this question, we believe that some of the men who advocate the extra college year are doing so because of selfish motives, believing that it will enable them to further control dental education, and they do not have the interest of the public or profession at heart.—*The International Journal,* July, 1921.

COMMENT.—The amount of time required for graduation in dentistry can rarely be determined by an individual School or College. Neither can it be adequately stated by any of the usual State Board requirements. At the present each State ought to be able to determine the character and extent of the knowledge and skill that the people need to at least safeguard their health, and should be competent to require this amount of skill and knowledge of every person offering such services to the public. Colleges and Schools at the present establish standards on past traditions for the sake of uniformity. Would it not be wiser for all if there were a general striving to emulate the highest attainments known as a basis of standards to be universally recognized? But perhaps we are not yet ready for such an advancement. We have yet a lot to learn before we can dogmatize on this matter.—EDITOR, *Register.*

